# Comparative transcriptomic analysis provides insights into the molecular basis underlying pre-harvest sprouting in rice

**DOI:** 10.1186/s12864-022-08998-4

**Published:** 2022-11-24

**Authors:** Dong Liu, Mingyang Zeng, Yan Wu, Yanli Du, Jianming Liu, Shaoqiang Luo, Yongjun Zeng

**Affiliations:** Key Laboratory of Crop Physiology, Ecology and Genetic Breeding, Ministry of Education, Jiangxi Agricultural University, Nanchang, 330045 China

**Keywords:** Rice, Pre-harvest sprouting, High-humidity stress, Transcriptomic analysis, Differentially expressed genes

## Abstract

**Background:**

Pre-harvest sprouting (PHS) is one of the most serious rice production constraints in areas where prolonged rainfall occurs during harvest. However, the molecular mechanisms of transcriptional regulation underlying PHS remain largely unknown.

**Results:**

In the current study, comparative transcriptome analyses were performed to characterize the similarities and differences between two rice varieties: PHS-sensitive Jiuxiangzhan (JXZ) and PHS-resistant Meixiangxinzhan (MXXZ). The physiological experimental results indicated that PHS causes a significant decrease in starch content and, in contrast, a significant increase in soluble sugar content and amylase activity. The extent of change in these physiological parameters in the sensitive variety JXZ was greater than that in the resistant variety MXXZ. A total of 9,602 DEGs were obtained from the transcriptome sequencing data, and 5,581 and 4,021 DEGs were identified in JXZ and MXXZ under high humidity conditions, respectively. The KEGG pathway enrichment analysis indicated that many DEGs under high humidity treatment were mainly linked to plant hormone signal transduction, carbon metabolism, starch and sucrose metabolism, and phenylpropanoid biosynthesis. Furthermore, the number of upregulated genes involved in these pathways was much higher in JXZ than in MXXZ, while the number of downregulated genes was higher in MXXZ than in JXZ. These results suggest that the physiological and biochemical processes of these pathways are more active in the PHS-sensitive JXZ than in the PHS-resistant MXXZ.

**Conclusion:**

Based on these results, we inferred that PHS in rice results from altered phytohormone regulation, more active carbon metabolism and energy production, and enhanced phenylpropanoid biosynthesis. Our study provides a theoretical foundation for further elucidation of the complex regulatory mechanism of PHS in rice and the molecular breeding of PHS-resistant rice varieties.

**Supplementary Information:**

The online version contains supplementary material available at 10.1186/s12864-022-08998-4.

## Background

Pre-harvest sprouting (PHS) is the phenomenon of germinating grains in mature cereal spikes before harvest under climatic conditions of increased rainfall and humidity. PHS in rice is an adverse biological phenomenon that results in weather-dependent reductions in the yield and quality of grain worldwide [1, 2]. In South China alone, severe PHS damages more than 6% of the planting area of conventional rice and as much as 20% of the planting area of hybrid rice [3]. Therefore, it is important to investigate the physiological and molecular mechanisms underlying and regulating PHS susceptibility in rice.

Seed dormancy is a crucial trait of economic importance in rice production because of its association with PHS, which occurs when rice is exposed to excessive moisture in the field. In general, inadequate dormancy is the main determinant of PHS [1, 4]. Both endogenous and exogenous factors, including phytohormones, light, and temperature, modulate seed dormancy through complex pathways [5, 6]. Extensive studies have shown that abscisic acid (ABA) and gibberellin acid (GA) are the major phytohormones regulating seed dormancy [4, 7–9]. ABA is required for seed dormancy induction, while GA is essential for breaking seed dormancy [8, 10]. The balance between the spatiotemporal concentration and accumulation of the two phytohormones and their respective signal pathway activation is especially crucial in controlling both the induction and breaking of dormancy [5]. In rice, ABA regulates seed dormancy mainly through its effect on the ratio of ABA to GA [11, 12]. In addition, auxin indirectly regulates seed dormancy via interaction with ABA signalling and ABI3 activation [13]. The ambient light and temperature environmental variations also affect seed dormancy by disturbing the balance between ABA and GA levels in cereal crops [14, 15].

It has been reported that endospermic carbohydrates serve as an important source of energy for seed germination and control seed dormancy [16, 17]. On the one hand, starch constitutes endosperm tissue and is the main energy source for the seed germination of cereal plants. When rice seeds absorb adequate water, α-amylase is activated in the aleurone layer and degrades starch into soluble sugars in the endosperm. These soluble sugars are then transported into the embryo to be used in cellular respiration. However, seed dormancy is also affected by endosperm tissue components and factors. It was demonstrated previously that barley mutants with low rates of starch biosynthesis in the developing endosperm have low grain dormancy and high susceptibility to PHS [18]. Excess soluble sugars in cereal grains can decrease grain sensitivity to ABA [19]. In contrast, sugar depletion can increase GA sensitivity, resulting in α-amylase activation and starch degradation [20, 21]. These previous studies have demonstrated that carbohydrate metabolism and phytohormones are inextricably linked to PHS.

Since PHS causes yield losses, adversely impacts grain quality, and leads to a large economic loss in rice production worldwide, significant attention has been devoted to investigating the molecular mechanisms of PHS tolerance for breeding PHS-resistant rice varieties. Numerous studies in different plant species have concluded that PHS resistance is highly quantitative and controlled by many quantitative trait loci [22–24]. In rice, accumulating studies indicate that many characterized genes that participate in PHS regulation are mainly involved in ABA biosynthesis, catabolism, and signalling. *Osaba1*, the first identified PHS-related mutant in rice, exhibits a strong viviparous phenotype due to an insertion resulting in a loss of function of the *OsABA1* gene that leads to decreased ABA biosynthesis [25]. Afterwards, a series of T-DNA insertion *phs* mutants were isolated from a rice mutagenesis library, and some of these mutants were characterized to further investigate the molecular mechanisms of PHS. Among them, four mutants (*phs1*, *phs2*, *phs3*, and *phs4*) were mutations in genes encoding OsPDS, OsZDS, OsCRTISO, and β-OsLCY, which are required for ABA carotenoid precursor biosynthesis [26]. The PHS phenotype of rice *phs8* mutants is caused by a T-DNA insertion in the gene *OsISA1* encoding a major starch biosynthesis enzyme involved in the regulation of amylopectin biosynthesis. Further studies demonstrated that impaired endosperm starch biosynthesis directly affects the ABA responsiveness of *phs8* rice seeds, eventually leading to PHS [16]. Using activation-tagging technology, Xu et al. (2019) recently identified a rice mutant *pre-harvest sprouting 9* (*phs9-D*) that exhibited a severe PHS phenotype. *PHS9*, which encodes a higher plant unique CC-type GRX, combines with OsGAP to disrupt ABA signalling and negatively regulates PHS in rice [27]. Although PHS in rice appears to be controlled by some specific genes with major effects, very little is known about the underlying regulatory mechanisms and interactions during the PHS response.

In this study, two rice varieties, a PHS-sensitive variety (JXZ) and a PHS-resistant variety (MXXZ), were used as experimental materials. We aimed to evaluate and compare their physiological and transcriptomic responses to high humidity. The results indicated that the promotion of PHS in rice resulted from altered phytohormone regulation, more active carbon metabolism and energy production, and enhanced phenylpropanoid biosynthesis. Our results provide an important foundation for understanding the molecular mechanisms of transcriptional regulation of PHS in rice and useful information on the selection and breeding of PHS-resistant varieties cultivated in a region with high rainfall and humidity.

## Results

### The indica rice varieties Jiuxiangzhan (JXZ) and Meixiangxinzhan (MXXZ) exhibit differences in PHS phenotypes

**Fig. 1:**
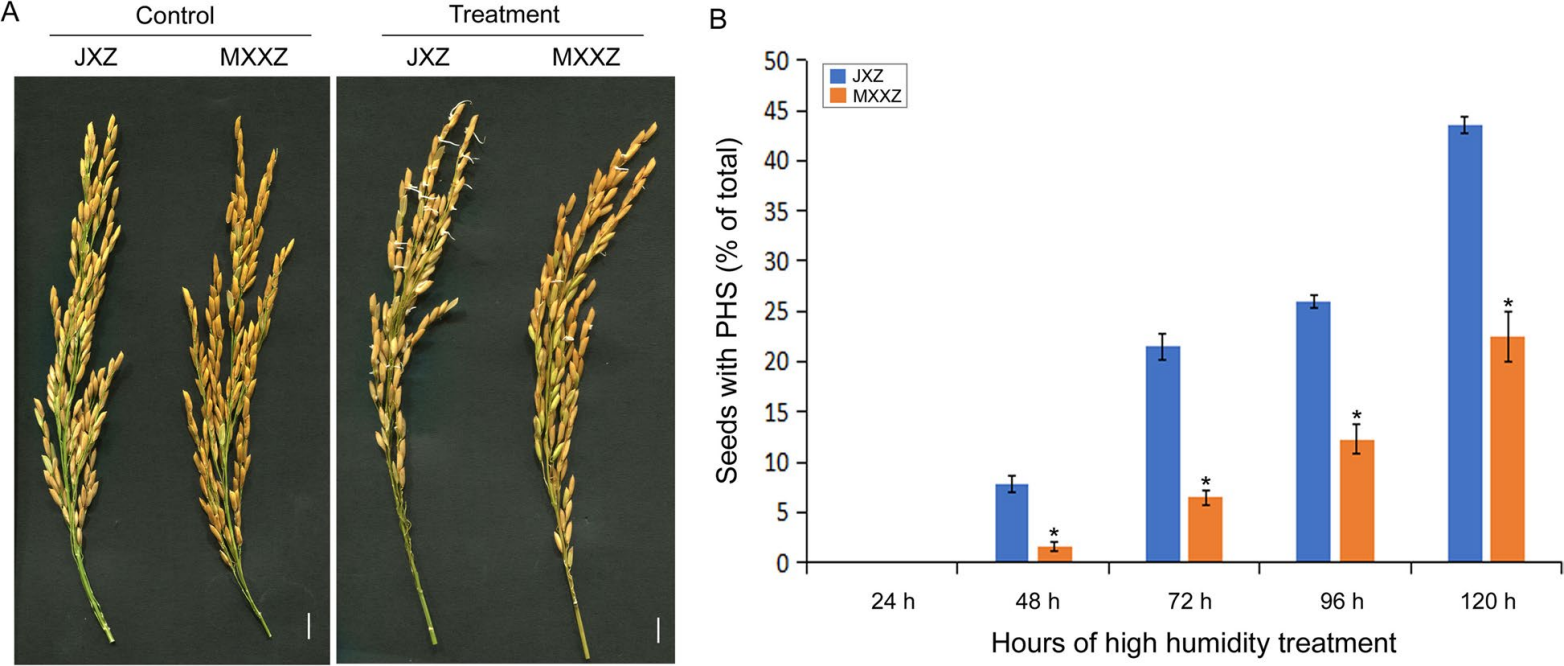
Comparison of the pre-harvest sprouting (PHS) vigour of JXZ and MXXZ under high humidity conditions. **A** The PHS phenotype of JXZ and MXXZ varieties after treatment with high humidity for 120 h. Scale bar = 1.0 cm. **B** Time-course PHS rates of JXZ and MXXZ varieties. All data represent the means ± standard errors of three replicated experiments (three biological replicates were performed for each experiment). Values with asterisks indicate a significant difference at the *P* < 0.05 level between the JXZ and MXXZ varieties

In our previous experiments on rice cultivation technologies, we accidentally identified a significant difference in the PHS phenotype between the two rice varieties, i.e., JXZ and MXXZ, under excessive rainfall conditions. The results reported in the current study were consistent with those previous observations (Fig. 1). High-humidity treatment in the artificial climate chamber promoted seed germination (no PHS was observed under the control conditions), and the germination rate of JXZ was significantly higher than that of MXXZ (Fig. 1). No germination was observed during the 24 h of the test. 48 h after starting treatment, JXZ had 7.8% seed germination compared to 1.6% in MXXZ. When the treatment period was extended to 120 h, JXZ was similarly more sensitive to PHS than MXXZ, with seed germination rates of 43.6% and 22.4% for JXZ and MXXZ, respectively (Fig. 1). These results confirmed that JXZ was much more sensitive to PHS than MXXZ. We next selected JXZ and MXXZ seeds during the 48 h germination point under high humidity treatment and control conditions to investigate the PHS mechanisms in rice. There are two main reasons for this. First, the purpose of our study is to investigate the physiological and molecular changes that occur at the initial stage of the PHS in rice. Second, the results of statistical analysis showed that significant differences were present in the seed germination between JXZ and MXXZ when the treatment time was extended to 48 h while no germination was observed during the 24 h of the high-humidity treatment.

### Starch content, soluble sugar content, and amylase activity changes under high humidity conditions in the two rice varieties

**Fig. 2:**
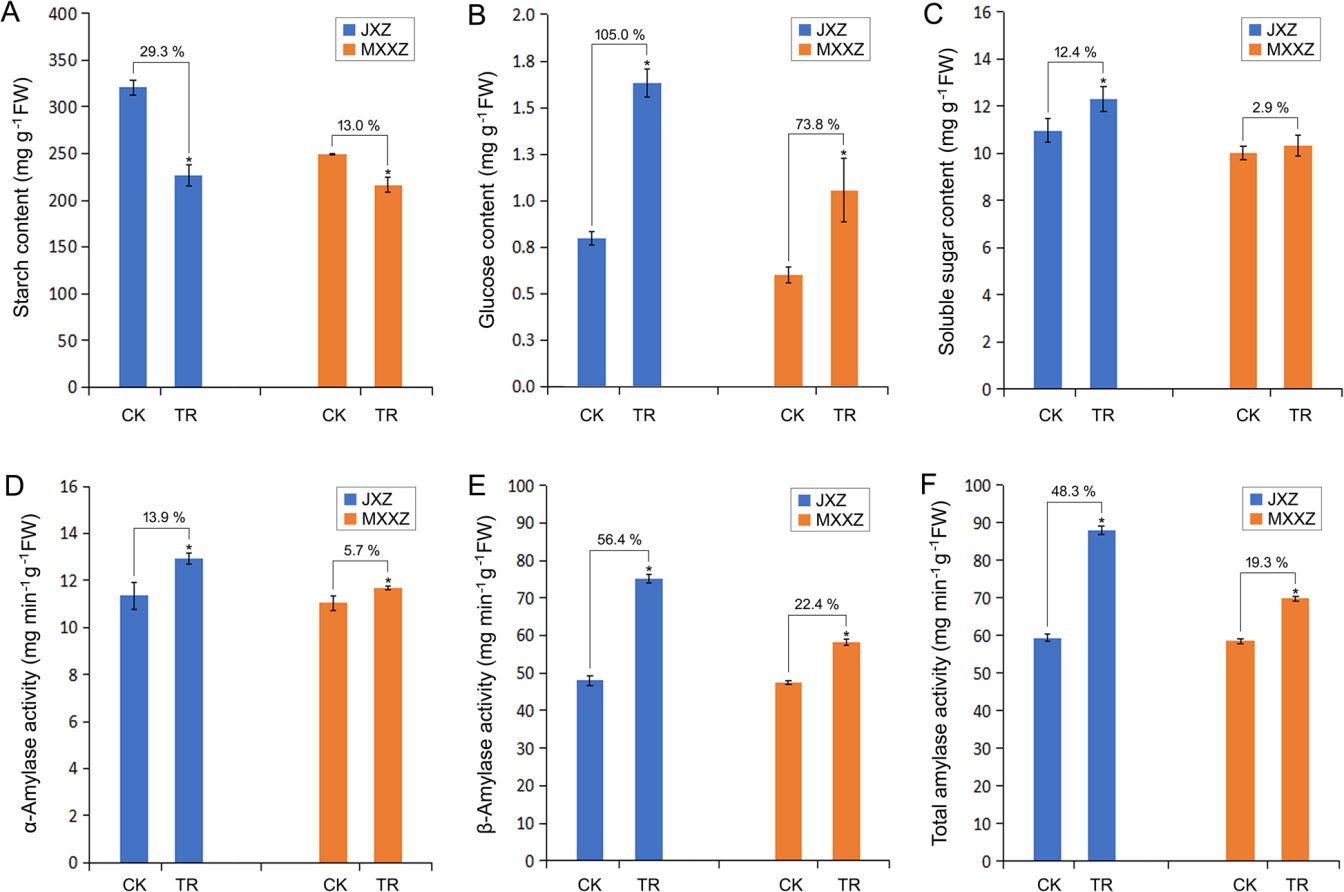
Comparative changes in the physiological parameters in seeds of two rice varieties (JXZ and MXXZ) upon PHS. **A** Starch content. **B** Glucose content. **C** Soluble sugar content. **D** α-Amylase activity. **E** β-Amylase activity. **F** Total amylase activity. CK indicates control, and TR indicates high humidity treatment. All data represent the means ± standard errors of three replicated experiments (three biological replicates were performed for each experiment). Values with asterisks indicate a significant difference at the *P* < 0.05 level between the treatment and control groups

To better understand the physiological mechanisms associated with PHS resistance in the rice varieties evaluated, we first examined the changes in the starch, glucose, and soluble sugar contents in seeds. Compared to the control, the high humidity treatment significantly reduced the starch content by 29.3% for JXZ and only 13.0% for MXXZ (Fig. 2A). Under high humidity conditions, the glucose content of JXZ increased by 105.0%, while that of MXXZ increased by only 73.8% compared to the control (Fig. 2B). The soluble sugar content of JXZ increased by 12.4% compared to the control, while no significant changes were observed in MXXZ (Fig. 2C). The decrease in starch content might be due to its degradation by the action of amylolytic enzymes. We therefore measured the changes in amylase activity in the seeds of the two rice varieties. In the control group, there were no significant differences in the activities of α-amylase, β-amylase and total amylase between the two varieties. Although the activities of these enzymes were dramatically induced in both rice varieties under high humidity conditions, the extent of induction in MXXZ was not as high as that in JXZ (the activities of α-amylase, β-amylase and total amylase in JXZ respectively increased by 13.9%, 56.4% and 48.3%, while that of MXXZ respectively increased by only 5.7%, 22.4% and 19.3%; Fig. 2D-F). Therefore, the increase in amylase activity of the sensitive to PHS JXZ was higher compared to MXXZ under high humidity conditions, providing a potential causal effect of increased amylase activity and the PHS phenotype.

### Differentially expressed genes (DEGs) between JXZ and MXXZ under high humidity conditions

**Fig. 3:**
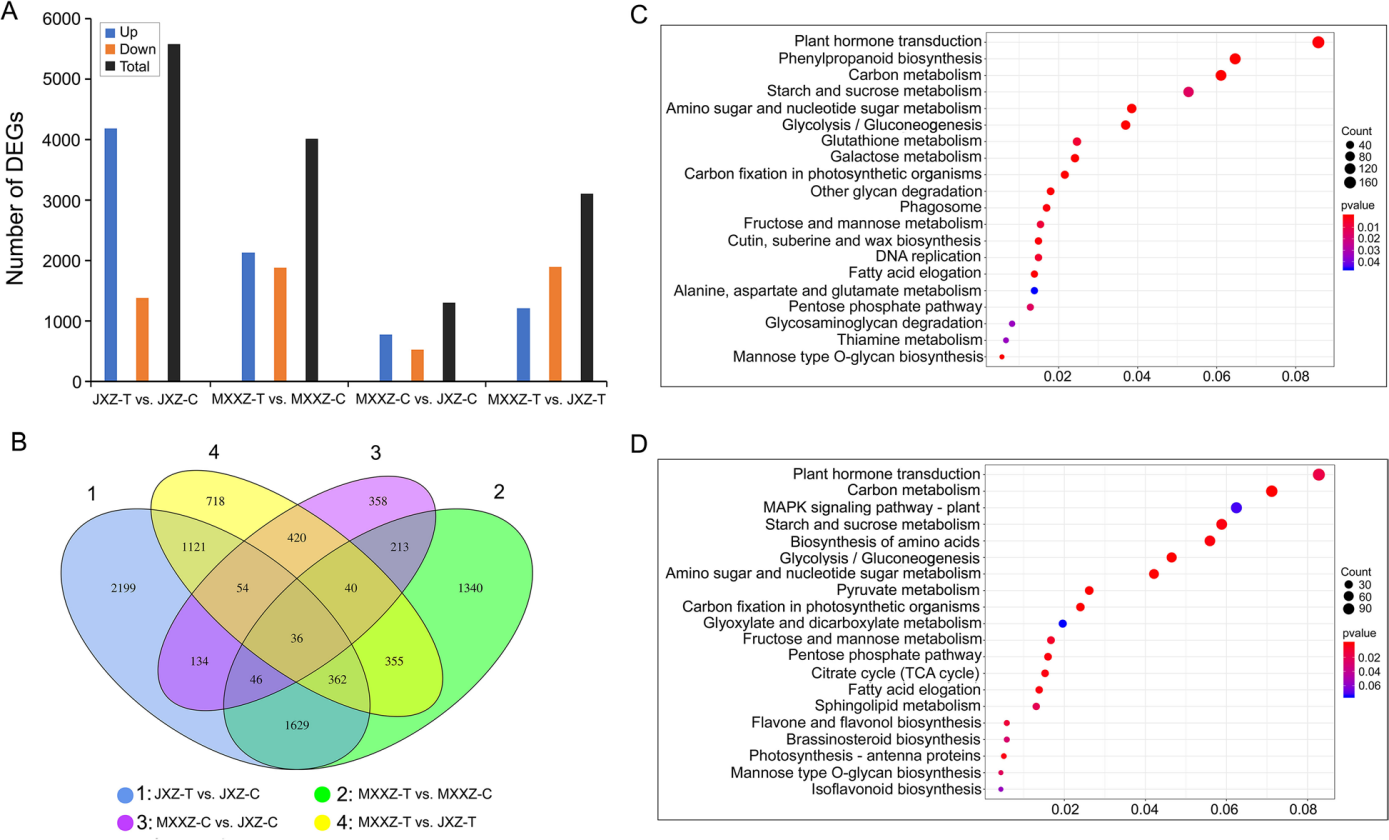
Analysis of DEGs in seeds of two rice varieties (JXZ and MXXZ) in response to high humidity. **A** The number of DEGs in different comparison groups. **B** Venn diagrams illustrate the overlap of DEGs among different comparisons. **C** Scatter plot of the top 20 KEGG pathways for the DEGs in JXZ-T vs. JXZ-C. **D** Scatter plot of the top 20 KEGG pathways for the DEGs in MXXZ-T vs. MXXZ-C. The x-axis indicates the gene ratio of DEGs belonging to the corresponding pathway, and the y-axis represents the enriched pathways. The size of each point indicates the number of DEGs, and the colour represents the range of the corrected *P* value. "JXZ-C," "JXZ-T," "MXXZ-C," and "MXXZ-T" indicate the control (C) and PHS-induced (T) samples of the JXZ and MXXZ varieties, respectively

To gain a broader understanding of the PHS-induced changes at the transcriptome level in rice, RNA-Seq was performed. The control and PHS-induced seeds of JXZ and MXXZ varieties were used (named JXZ-C or MXXZ-C for control samples and JXZ-T or MXXZ-T for PHS-induced samples). To further identify the genes most closely correlated with the differential PHS phenotypes of the two varieties, differentially expressed genes (DEGs) were obtained by comparing gene expression levels in JXZ and MXXZ. We evaluated four comparison groups: JXZ-T vs. JXZ-C, MXXZ-T vs. MXXZ-C, MXXZ-C vs. JXZ-C, and MXXZ-T vs. JXZ-T. According to the sequencing data, 5,581 (5,459 were annotated) and 4,021 (3,876 were annotated) DEGs were identified in PHS-sensitive (JXZ-T vs. JXZ-C) and PHS-resistant (MXXZ-T vs. MXXZ-C) varieties, respectively (Fig. 3A; Table S1, S2). Among them, a total of 4,189 (4,149 were annotated) DEGs showed upregulation, and 1,392 (1,310 were annotated) DEGs showed downregulation in the PHS-sensitive variety (JXZ-T vs. JXZ-C), whereas 2,132 (2,050 were annotated) DEGs showed upregulation, and 1,889 (1,826 were annotated) DEGs showed downregulation in the PHS-tolerant variety (MXXZ-T vs. MXXZ-C; Fig. 3A; Table S1, S2). Notably, consistent with the physiological observations, more upregulated genes were identified in PHS-sensitive JXZ than in PHS-resistant MXXZ. In contrast, more downregulated genes were identified in PHS-resistant MXXZ than in PHS-sensitive JXZ (Fig. 3A). A total of 1,301 (1,157 were annotated) and 3,106 (2,958 were annotated) DEGs were identified when comparing MXXZ-C vs. JXZ-C and MXXZ-T vs. JXZ-T, respectively (Fig. 3A; Table S3, S4). Moreover, a Venn diagram of the DEG analysis indicated that 2073 genes were shared between JXZ-T vs. JXZ-C and MXXZ-T vs. MXXZ-C (Fig. 3B).

### KEGG and Gene Ontology (GO) enrichment analysis

We further determined whether the PHS-induced DEGs in the two varieties were involved in specific pathways by querying the KEGG public database. The DEGs were categorized into 134 and 131 KEGG pathways in JXZ and MXXZ, respectively (Table S5, S6). Among the top 20 KEGG pathways, 10 pathways were common in both PHS-sensitive and PHS-resistant varieties (Fig. 3C, D; Table 1). These included 3 major pathways (plant hormone signal transduction, carbon metabolism, and starch and sucrose metabolism) and 7 other pathways (amino sugar and nucleotide sugar metabolism, glycolysis/gluconeogenesis, carbon fixation in photosynthetic organisms, fructose and mannose metabolism, fatty acid elongation, pentose phosphate pathway, and mannose type O-glycan biosynthesis). This is indicative of common mechanisms existing in both PHS-sensitive and PHS-resistant varieties. Notably, the total number of DEGs for each pathway was much higher in JXZ than in MXXZ (Table 1). In addition, DEGs associated with phenylpropanoid biosynthesis, glutathione metabolism, galactose metabolism, other glycan degradation, phagosome, cutin, suberin and wax biosynthesis, DNA replication, alanine, aspartate and glutamate metabolism, glycosaminoglycan degradation, and thiamine metabolism were enriched in JXZ (Fig. 3C). MXXZ DEGs, on the other hand, were enriched in pathways involving the MAPK signalling pathway, including plant, biosynthesis of amino acids, pyruvate metabolism, glyoxylate, dicarboxylate metabolism, citrate cycle, sphingolipid metabolism, flavone, flavonol biosynthesis, brassinosteroid biosynthesis, photosynthesis, antenna proteins, and isoflavonoid biosynthesis (Fig. 3D). Notably, a larger number of DEGs associated with phenylpropanoid biosynthesis were specifically enriched in JXZ (Fig. 3C). These results indicated that common and genotype-specific PHS mechanisms exist in both sensitive and resistant varieties.

**Table 1:** Top 20 KEGG enrichment analysis of DEGs in JXZ-T vs. JXZ-C and MXXZ-T vs. MXXZ-C

**Pathway term**	**Term ID**	**Gene number**		
		**Up**	**Down**	**Total**
**JXZ-T vs. JXZ-C**				
Plant hormone signal transduction	ko 04,075	143	24	167
Phenylpropanoid biosynthesis	ko 00,940	120	6	126
Carbon metabolism	ko 01,200	101	18	119
Starch and sucrose metabolism	ko 00,500	92	11	103
Amino sugar and nucleotide sugar metabolism	ko 00,520	67	8	75
Glycolysis / gluconeogenesis	ko 00,010	62	10	72
Glutathione metabolism	ko 00,480	39	9	48
Galactose metabolism	ko 00,052	37	10	47
Carbon fixation in photosynthetic organisms	ko 00,710	36	6	42
Other glycan degradation	ko 00,511	33	2	35
Phagosome	ko 04,145	32	1	33
Fructose and mannose metabolism	ko 00,051	26	4	30
Cutin, suberine and wax biosynthesis	ko 00,073	26	3	29
DNA replication	ko 03,030	22	7	29
Fatty acid elongation	ko 00,062	25	2	27
Alanine, aspartate and glutamate metabolism	ko 00,250	22	5	27
Pentose phosphate pathway	ko 00,030	19	6	25
Glycosaminoglycan degradation	ko 00,531	15	1	16
Thiamine metabolism	ko 00,730	8	5	13
Mannose type O-glycan biosynthesis	ko 00,515	11	0	11
**MXXZ-T vs. MXXZ-C**				
Plant hormone signal transduction	ko 04,075	78	36	114
Carbon metabolism	ko 01,200	60	38	98
MAPK signaling pathway—plant	ko 04,016	47	39	86
Starch and sucrose metabolism	ko 00,500	55	26	81
Biosynthesis of amino acids	ko 01,230	49	28	77
Glycolysis / Gluconeogenesis	ko 00,010	51	13	64
Amino sugar and nucleotide sugar metabolism	ko 00,520	47	11	58
Pyruvate metabolism	ko 00,620	25	11	36
Carbon fixation in photosynthetic organisms	ko 00,710	20	13	33
Glyoxylate and dicarboxylate metabolism	ko 00,630	12	15	27
Fructose and mannose metabolism	ko 00,051	18	5	23
Pentose phosphate pathway	ko 00,030	15	7	22
Citrate cycle (TCA cycle)	ko 00,020	11	10	21
Fatty acid elongation	ko 00,062	16	3	19
Sphingolipid metabolism	ko 00,600	14	4	18
Flavone and flavonol biosynthesis	ko 00,944	4	4	8
Brassinosteroid biosynthesis	ko 00,905	7	1	8
Photosynthesis—antenna proteins	ko 00,196	0	7	7
Mannose type O-glycan biosynthesis	ko 00,515	6	0	6
Isoflavonoid biosynthesis	ko 00,943	2	4	6

“JXZ-C”, “JXZ-T”, “MXXZ-C” and “MXXZ-T” indicate the control (C) and PHS-induced (T) samples of the JXZ and MXXZ varieties, respectively

**Fig. 4:**
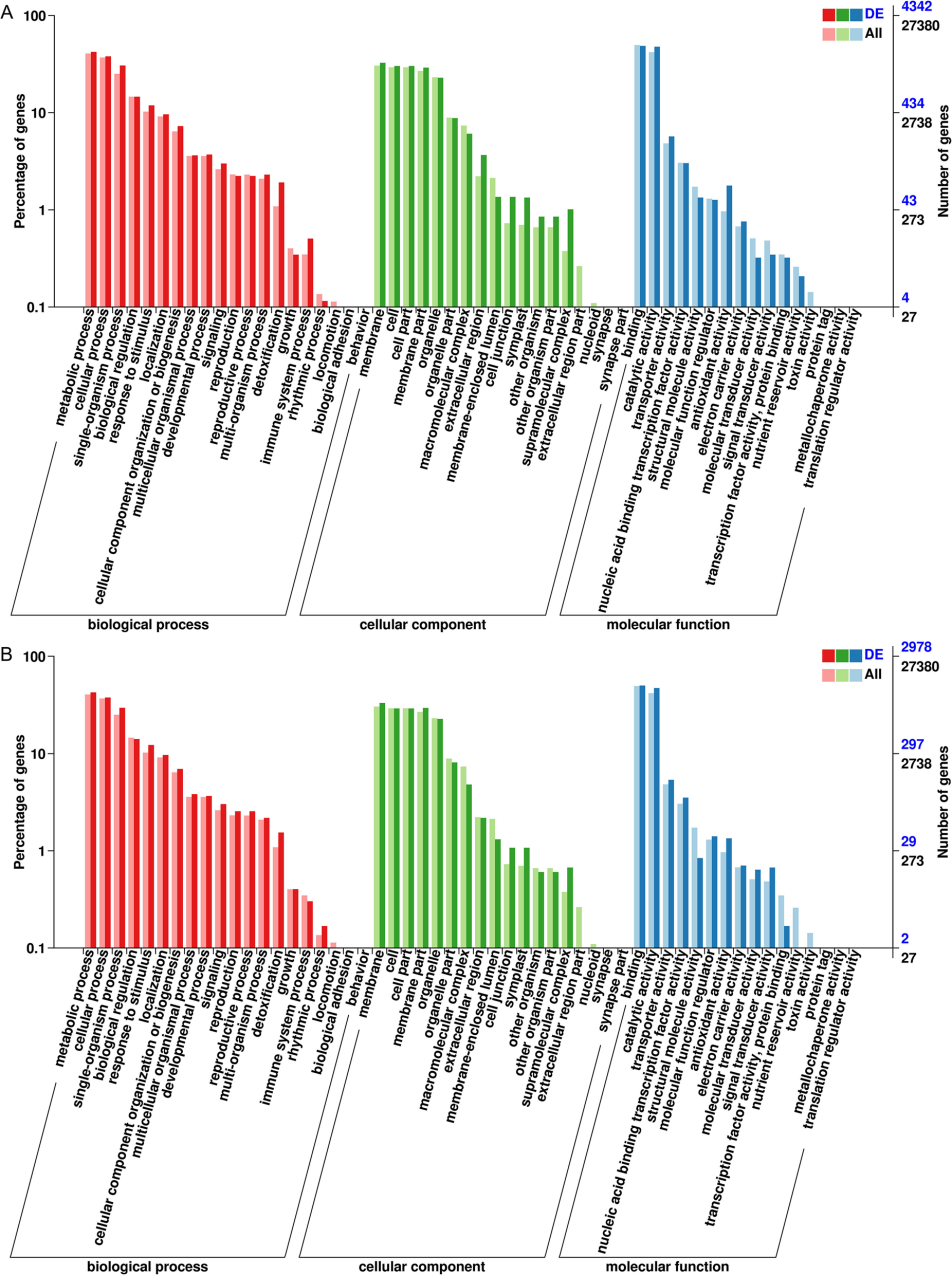
Gene ontology classification of biological process, cellular component, and molecular function of the DEGs from the two rice varieties (JXZ and MXXZ) in response to high humidity. **A** GO terms for DEGs overrepresented in JXZ. **B** GO terms for DEGs overrepresented in MXXZ. “DE” and “All” indicate the gene enrichment of each secondary GO terms under the background of differentially expressed unigenes and all unigenes, respectively

Gene Ontology (GO) was also used to classify the functions of DEGs identified in the pairwise comparison of JXZ-T vs. JXZ-C and MXXZ-T vs. MXXZ-C. DEGs were assigned to three primary GO categories: biological process, cellular component, and molecular function. A total of 19,786 and 13,419 DEGs from JXZ and MXXZ, respectively, were assigned GO terms (Table S7, S8). Among the 19,786 DEGs in JXZ, 7,562 DEGs (6100 upregulated and 1,462 downregulated) were assigned to the biological process category, 7,396 DEGs (5892 upregulated and 1,504 downregulated) were assigned to the cellular component category, and 4,842 DEGs (3888 upregulated and 954 downregulated) were assigned to the molecular function category (Table S7). Among the 13,419 DEGs from MXXZ, 5,169 DEGs (2,937 upregulated and 2,232 downregulated) were assigned to the biological process category, 4,899 DEGs (2,555 upregulated and 2,344 downregulated) were assigned to the cellular component category and 3,351 DEGs (1,923 upregulated and 1,428 downregulated) were assigned to the molecular function category (Table S8). Within the biological process category, overrepresented DEGs corresponded to metabolic processes (1,833 in JXZ and 1,271 in MXXZ), cellular processes (1649 in JXZ and 1,126 in MXXZ), and single-organism processes (1,326 in JXZ and 883 in MXXZ; Fig. 4; Table S7, S8). Within the cellular component category, overrepresented DEGs corresponded to membranes (1,416 in JXZ and 989 in MXXZ), cells (1,311 in JXZ and 869 in MXXZ), cell parts (1,311 in JXZ and 869 in MXXZ), membrane parts (1,262 in JXZ and 880 in MXXZ) and organelles (991 in JXZ and 679 in MXXZ; Fig. 4; Table S7, S8). Sixteen GO functional groups were assigned to the molecular function category, among which binding (2,114 in JXZ and 1,498 in MXXZ) and catalytic activity (2,071 in JXZ and 1,412 in MXXZ) were the most overrepresented in the two varieties (Fig. 4; Table S7, S8).

### DEGs associated with phytohormone metabolism and signalling

**Fig. 5:**
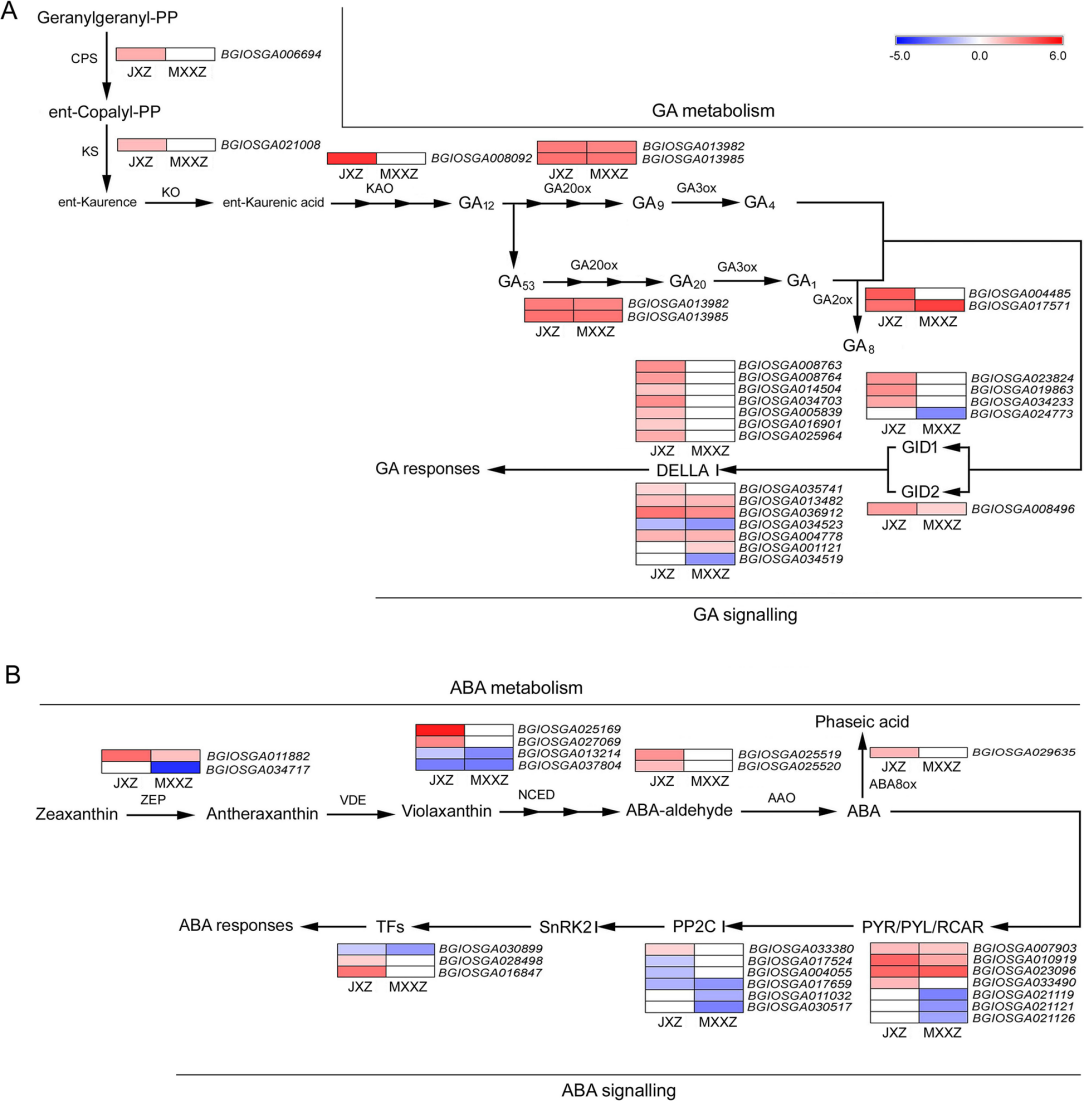
Heatmap diagram showing the DEGs involved in the metabolism and signalling of GA (**A**) and ABA (**B**) in JXZ and MXXZ under control and high humidity conditions. DEGs were analysed in two comparisons, JXZ-T vs. JXZ-C (JXZ) and MXXZ-T vs. MXXZ-C (MXXZ). "JXZ-C," "JXZ-T," “MXXZ-C,” and “MXXZ-T” indicate the control (C) and PHS-induced (T) samples of the JXZ and MXXZ varieties, respectively. The colour gradient shows the relative expression levels of DEGs from low (blue indicates downregulation) to high (red indicates upregulation). The numerical values of the blue-to-red gradient bar represent the log_2_ fold change in expression for the high humidity treatment relative to the control

In the KEGG analysis, "plant hormone signal transduction" was the most enriched pathway, with 167 DEGs in JXZ and 114 DEGs in MXXZ (Fig. 3C, D; Table S5, S6). Since GA and ABA are the major phytohormones that regulate seed dormancy and germination, DEGs involved in their metabolism and signalling were further investigated (Fig. 5; Table S9). Seven DEGs encoding GA metabolic enzymes were identified, including *CPS*, *KS*, *KAO*, *GA20ox*, and *GA2ox*. Three genes encoding CPS (*BGIOSGA006694*), KS (*BGIOSGA021008*), and KAO (*BGIOSGA008092*) were significantly upregulated in JXZ, while they remained unchanged in MXXZ. Genes encoding GA20ox (*BGIOSGA013982* and *BGIOSGA013985*) exhibited significant upregulation in both varieties. Two genes encoding GA2ox (*BGIOSGA004485* and *BGIOSGA017571*) were upregulated in JXZ, while only one gene (*BGIOSGA017571*) was upregulated in MXXZ. Furthermore, numerous genes associated with GA signal transduction were differentially expressed in response to PHS treatments in the two varieties (Fig. 5; Table S9). Specifically, four genes encoding gibberellin receptors, *GID1a-1* (*BGIOSGA023824*), *GID1b-1* (*BGIOSGA019863*), *GID1c-like isoform X2* (*BGIOSGA034233*) and *GID2* (*BGIOSGA008496*), were all significantly upregulated in JXZ. Among these, only *GID2* was upregulated in MXXZ. Nevertheless, its transcription level was higher in JXZ than in MXXZ. The gibberellin receptor GID1a-2 (*BGIOSGA024773*) gene was also downregulated in MXXZ. Moreover, the number of upregulated genes encoding DELLA proteins was much higher in JXZ than in MXXZ. In contrast, the number of downregulated DELLA genes was higher in MXXZ than in JXZ. In JXZ, eleven genes encoding DELLA proteins were upregulated, and only one gene (*BGIOSGA034523*) was downregulated. Four out of six genes (*BGIOSGA013482*, *BGIOSGA036912, BGIOSGA004778* and *BGIOSGA001121*) encoding DELLA proteins were upregulated, while two genes (*BGIOSGA034523* and *BGIOSGA034519*) were downregulated in MXXZ. Among these differentially expressed DELLA genes, four (*BGIOSGA013482*, *BGIOSGA036912*, *BGIOSGA034523* and *BGIOSGA004778*) were common in both varieties.

Genes related to ABA biosynthesis and signalling were also identified. Several DEGs encoding ABA metabolic enzymes, including *ZEP*, *NCED*, *AAO*, and *ABA8ox*, were identified (Fig. 5; Table S9). Five genes encoding ZEP (*BGIOSGA011882*), NCED (*BGIOSGA025169* and *BGIOSGA027069*), and AAO (*BGIOSGA025519* and *BGIOSGA025520*) were upregulated, while two genes encoding NCED (*BGIOSGA013214* and *BGIOSGA037804*) were downregulated in JXZ. Three common genes encoding ZEP (*BGIOSGA011882*) and NCED (*BGIOSGA013214* and *BGIOSGA037804*) showed the same trend in both varieties. Notably, a gene encoding ZEP (*BGIOSGA034717*) was downregulated in MXXZ and remained unchanged in JXZ. In addition, the gene encoding ABA8ox (*BGIOSGA029635*) was significantly upregulated in JXZ and remained unchanged in MXXZ. For the DEGs involved in ABA signal transduction, a total of sixteen DEGs were annotated (Fig. 5; Table S9). Among seven genes encoding ABA receptors PYR/PYL/RCAR, four genes (*BGIOSGA007903*, *BGIOSGA010919*, *BGIOSGA023096* and *BGIOSGA033490*) were upregulated in JXZ, of which three (*BGIOSGA007903*, *BGIOSGA010919* and *BGIOSGA023096*) were upregulated in MXXZ. Three additional PYR/PYL/RCAR genes (*BGIOSGA021119*, *BGIOSGA021121* and *BGIOSGA021126*) were downregulated in MXXZ. Most genes downstream of ABA receptors, such as *PP2C* and *ABI5* (encoding a bZIP transcription factor, *BGIOSGA030899*), were significantly downregulated in both varieties, whereas only one *PP2C* gene (*BGIOSGA033380*) was upregulated in JXZ. We also identified two important components (transcription factors) involved in ABA-mediated seed germination inhibition: HDA6 (*BGIOSGA028498*) and BBX21 (*BGIOSGA016847*). These genes were upregulated specifically in JXZ but unchanged in MXXZ.

### Analysis of DEGs involved in "carbon metabolism" and "starch and sucrose metabolism" pathways

**Fig. 6:**
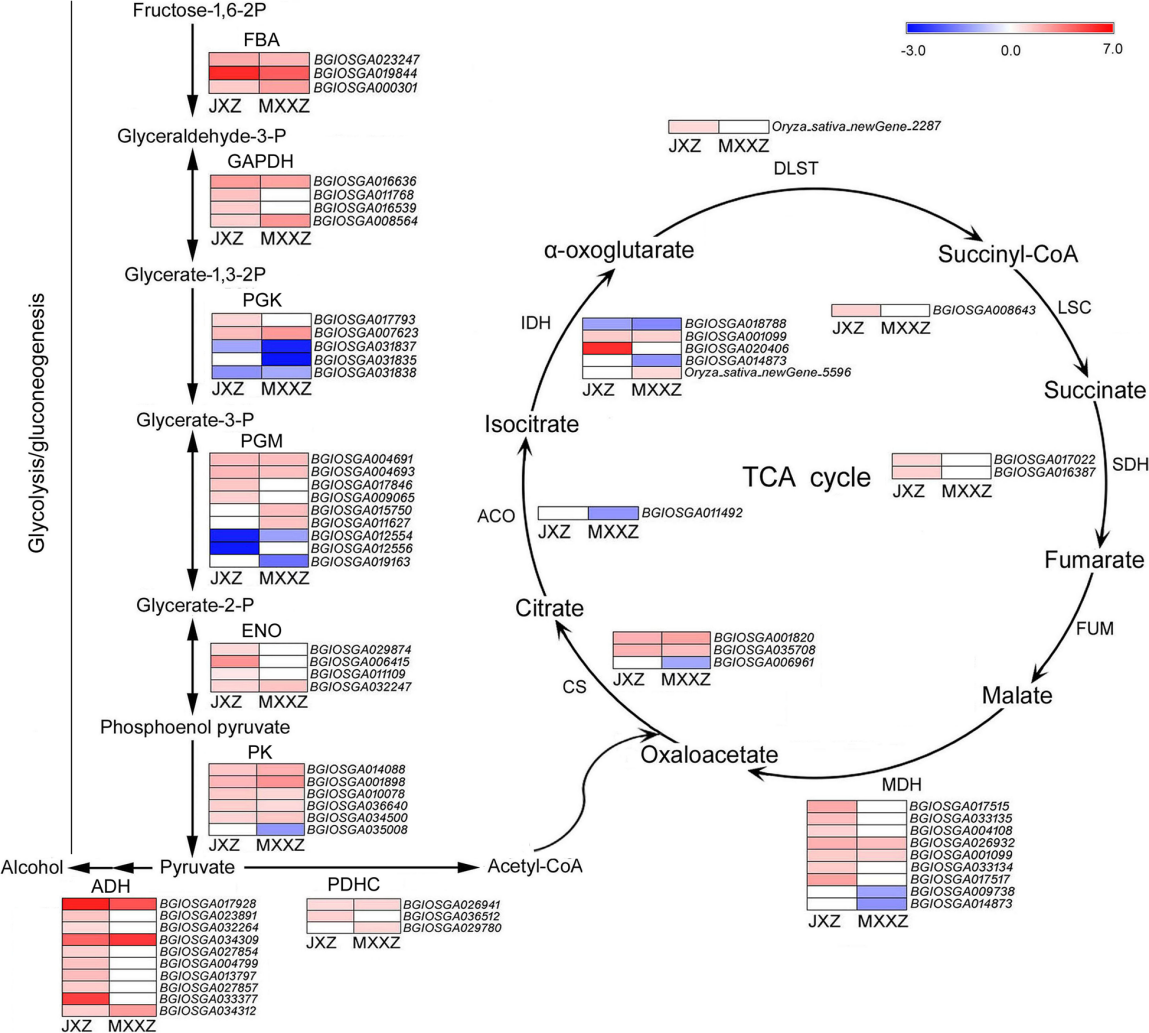
Heatmap diagram showing the DEGs involved in carbon metabolism (associated with energy production) in JXZ and MXXZ under control and high humidity conditions. The glycolysis/gluconeogenesis pathway connected by black straight arrow lines and citrate cycle (TCA cycle) pathway shown by the loop diagram. DEGs were analysed in two comparisons, JXZ-T vs. JXZ-C (JXZ) and MXXZ-T vs. MXXZ-C (MXXZ). "JXZ-C," "JXZ-T," "MXXZ-C," and "MXXZ-T" indicate the control (C) and PHS-induced (T) samples of the JXZ and MXXZ varieties, respectively. The colour gradient shows the relative expression levels of DEGs from low (blue indicates downregulation) to high (red indicates upregulation). The numerical values of the blue-to-red gradient bar represent the log_2_ fold change in expression for the high humidity treatment relative to the control

Carbon metabolism is an important process in higher plants for the generation of cellular energy and structural components. According to KEGG analysis, "carbon metabolism" was one of the most enriched pathways and contained 119 DEGs in JXZ and 98 DEGs in MXXZ (Fig. 3C, D; Table S5, S6). Our analysis showed that DEGs involved in carbon metabolism mainly included glycolysis/gluconeogenesis, the citrate cycle, starch and sucrose metabolism, and others. DEGs were identified as encoding enzymes related to glycolysis/gluconeogenesis, including FBA, GAPDH, PGK, PGM, ENO, and PK. Most genes involved in glycolysis/gluconeogenesis exhibited similar expression trends in both varieties (Fig. 6; Table S10). Although the number of downregulated DEGs encoding PGK was higher than that of upregulated genes in MXXZ and comparable to that of upregulated genes in JXZ, most DEGs encoding enzymes such as FBA, GAPDH, PGM, ENO, and PK were significantly upregulated in both varieties. After glycolysis, pyruvate molecules enter the citrate cycle (TCA cycle) to generate energy efficiency. Our results showed that nearly all genes encoding TCA cycle-associated enzymes, including PDHC, CS, IDH, DLST, LSC, SDH, and MDH, were upregulated in JXZ. However, most genes encoding these enzymes in MXXZ were downregulated or remained unchanged. Moreover, the genes encoding ADH involved in alcohol metabolism under anaerobic conditions were all significantly upregulated in JXZ, while most of them remained unchanged or upregulated (*BGIOSGA017928*) to a lower extent in MXXZ, and only two genes (*BGIOSGA034309* and *BGIOSGA034312*) were upregulated to a greater extent in MXXZ than in JXZ (Fig. 6; Table S10).

**Fig. 7:**
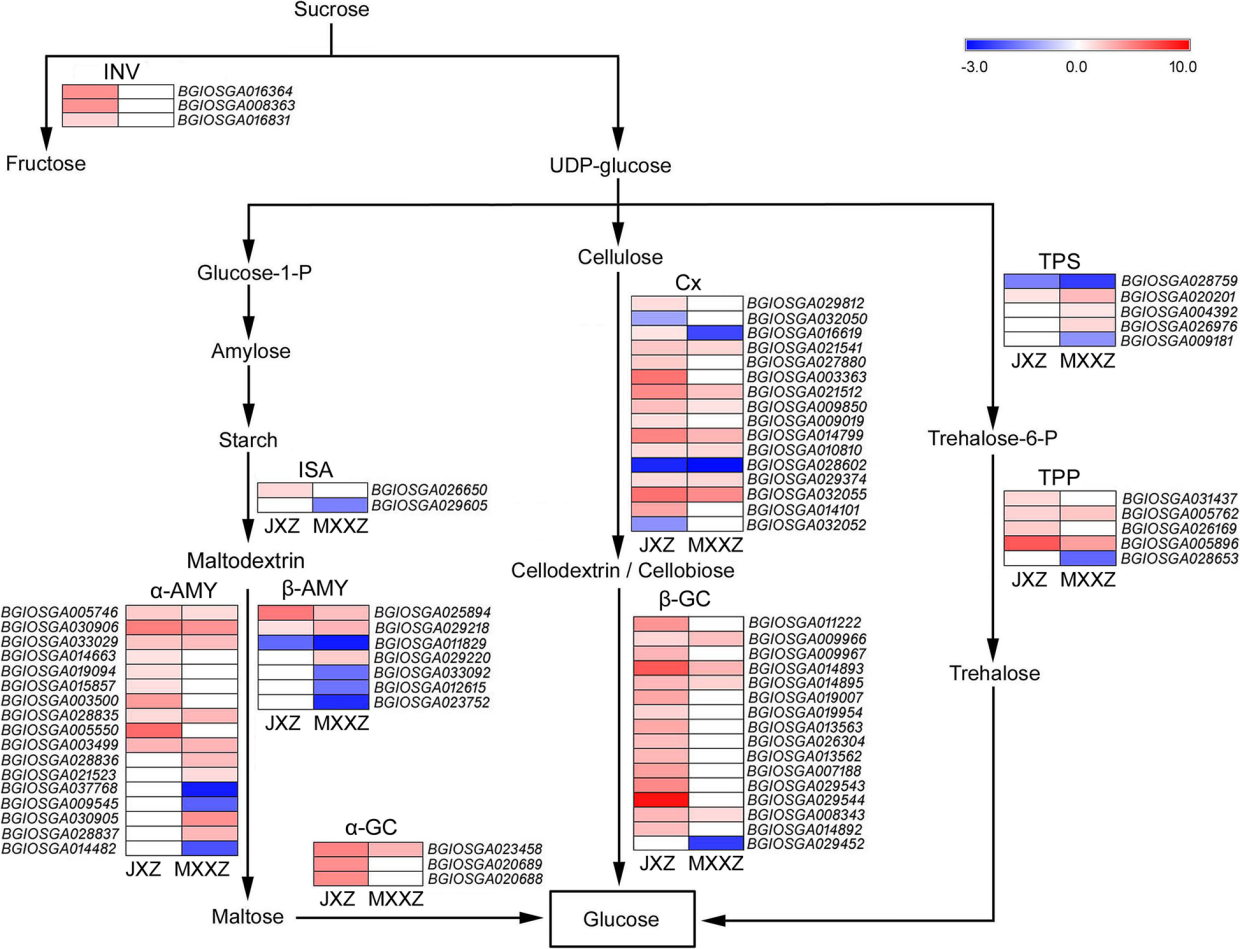
Heatmap diagram showing the DEGs involved in starch and sucrose metabolism in JXZ and MXXZ under control and high humidity conditions. DEGs were analysed in two comparisons, JXZ-T vs. JXZ-C (JXZ) and MXXZ-T vs. MXXZ-C (MXXZ). "JXZ-C," "JXZ-T," “MXXZ-C,” and “MXXZ-T” indicate the control (C) and PHS-induced (T) samples of the JXZ and MXXZ varieties, respectively. The colour gradient shows the relative expression levels of DEGs from low (blue indicates downregulation) to high (red indicates upregulation). The numerical values of the blue-to-red gradient bar represent the log_2_ fold change in expression for the high humidity treatment relative to the control

The starch and sucrose metabolism pathway-enriched DEGs were also analysed, with 103 and 81 DEGs identified in JXZ and MXXZ, respectively (Fig. 3C, D; Table S5, S6). Most of the DEGs involved in starch and sucrose metabolism were upregulated in both varieties, with a higher number of upregulated genes in JXZ than in MXXZ (Fig. 7; Table S11). Specifically, three genes (*BGIOSGA016364*, *BGIOSGA008363* and *BGIOSGA016831*) encoding INV were significantly upregulated in JXZ but remained unchanged in MXXZ. The total number of upregulated genes encoding the three categories of amylases (α-AMY, β-AMY, and ISA) in JXZ was greater than that in MXXZ. In contrast, the total number of downregulated genes was greater in MXXZ than in JXZ (Fig. 7; Table S11). Additionally, three genes (*BGIOSGA023458*, *BGIOSGA020689* and *BGIOSGA020688*) encoding α-GC were upregulated in JXZ, while only one shared gene (*BGIOSGA023458*) was upregulated in MXXZ. Genes encoding Cx and β-GC showed similar expression trends in both varieties; however, they increased to a much greater extent in JXZ than in MXXZ (Fig. 7; Table S11). In contrast with these results, more DEGs encoding TPS were identified in MXXZ than in JXZ. Three (*BGIOSGA020201*, *BGIOSGA004392* and *BGIOSGA026976*) of the five genes encoding TPS were upregulated, while two genes (*BGIOSGA028759* and *BGIOSGA009181*) were downregulated in MXXZ. Among these differentially expressed *TPS* genes, two (*BGIOSGA028759* and *BGIOSGA020201*) were common in both varieties (Fig. 7; Table S11). In addition, four genes (*BGIOSGA031437*, *BGIOSGA005762*, *BGIOSGA026169* and *BGIOSGA005896*) encoding TPP were all significantly upregulated in JXZ. In comparison, two (*BGIOSGA005762* and *BGIOSGA005896*) of the four shared genes encoding TPP were upregulated (the remaining two genes remained unchanged), and an additional gene (*BGIOSGA028653*) was downregulated in MXXZ (Fig. 7; Table S11).

### Analysis of DEGs involved in the "phenylpropanoid biosynthesis" pathway

**Fig. 8:**
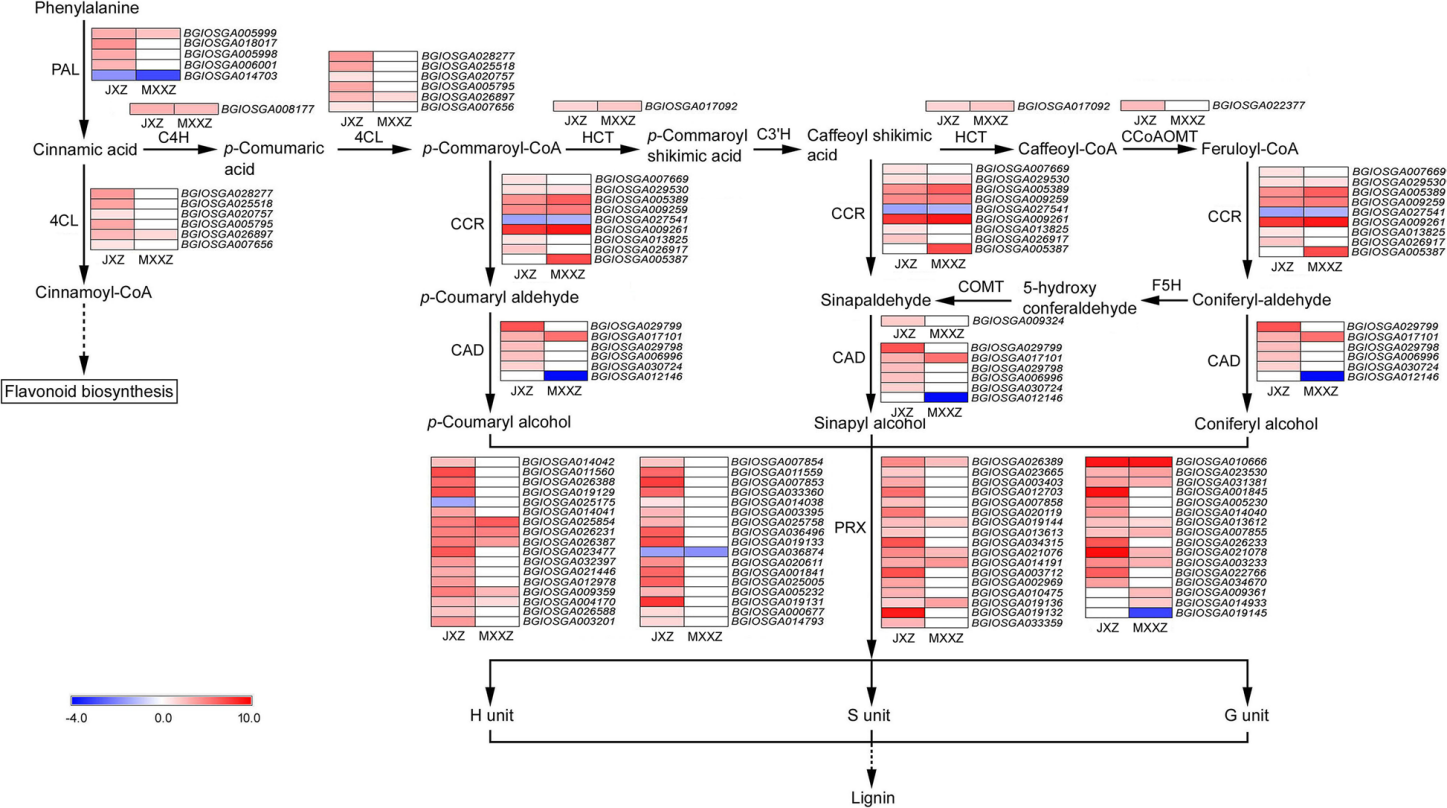
Heatmap diagram showing the DEGs involved in phenylpropanoid biosynthesis in JXZ and MXXZ under control and high humidity conditions. DEGs were analysed in two comparisons, JXZ-T vs. JXZ-C (JXZ) and MXXZ-T vs. MXXZ-C (MXXZ). “JXZ-C,” “JXZ-T,” “MXXZ-C,” and “MXXZ-T” indicate the control (C) and PHS-induced (T) samples of the JXZ and MXXZ varieties, respectively. The colour gradient shows the relative expression levels of DEGs from low (blue indicates downregulation) to high (red indicates upregulation). The numerical values of the blue-to-red gradient bar represent the log_2_ fold change in expression for the high humidity treatment relative to the control

The phenylpropanoid biosynthesis pathway was one of the most enriched KEGG pathways specific to JXZ (Fig. 3C). In this pathway, phenylalanine is converted to p-coumaroyl-CoA through successive catalytic steps by PAL, C4H, and 4CL. We found that most of the DEGs encoding these enzymes in both varieties were upregulated, and the number of upregulated genes in JXZ was much higher than that in MXXZ (Fig. 8; Table S12). The CCR and CAD enzymes are involved in the biosynthesis of *p*-coumaryl aldehyde and *p*-coumaryl alcohol, respectively. Almost all the DEGs encoding the CCR and CAD family members exhibited a significant PHS-inducible response in JXZ. Although these DEGs showed the same expression trends in MXXZ, the number of upregulated genes was much lower than that in JXZ, especially for *CAD* (Fig. 8; Table S12). In the pathway of sinapyl alcohol biosynthesis, *p*-commaroyl-CoA is converted into sinapyl alcohol through several enzymatic reactions. The enzymes involved are encoded by the genes *HCT*, *C3'H*, *CCoAOMT*, *F5H*, and *COMT*. Finally, the monolignols are derived from the three hydroxycinnamyl alcohols (*p*-coumaryl alcohol, sinapyl alcohol, and coniferyl alcohol) by PRX. In response to PHS, dramatic upregulation of most DEGs encoding these enzymes was observed in both varieties, especially in JXZ. Specifically, one shared DEG (*BGIOSGA017092*) encoding HCT showed the same trend (upregulated) in both JXZ and MXXZ. Two DEGs (*BGIOSGA022377* and *BGIOSGA009324*) encoding CCoAOMT and COMT were significantly upregulated in JXZ and remained unchanged in MXXZ (Fig. 8; Table S12). Moreover, many DEGs encoding PRX were differentially expressed in response to PHS in both varieties (64 DEGs in JXZ and 21 DEGs in MXXZ). The expression of DEGs mapped to PRX was increased in both the JXZ and MXXZ varieties; however, greater fold changes were observed in JXZ (Fig. 8; Table S12).

### Validation of RNA-Seq data by qRT‒PCR analysis

**Fig. 9:**
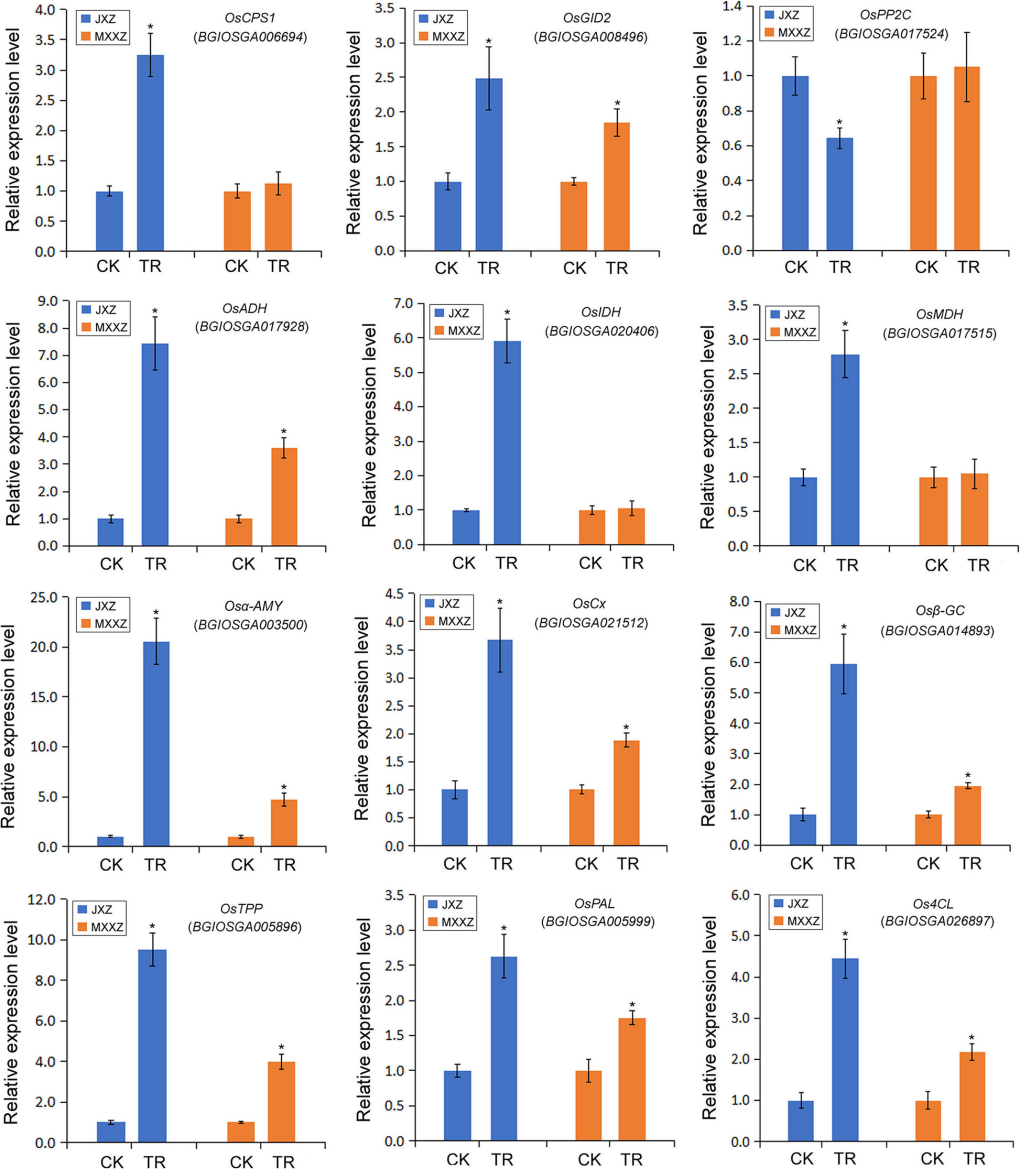
RNA-Seq results verification by real-time quantitative polymerase chain reaction (qRT‒PCR). The expression levels of each gene are shown as a ratio relative to the control (set to 1.0). CK indicates control, and TR indicates high humidity treatment. All data represent the means ± standard errors of three replicated experiments (three biological replicates were performed for each experiment). Values with asterisks indicate significant differences at the *P* < 0.05 level between the treatment (TR) and control (CK) groups

To further validate the accuracy of our transcriptome data, the gene expression profiles of twelve randomly selected DEGs were validated by qRT‒PCR. These DEGs included three genes involved in phytohormone metabolism signal transduction, namely, *OsCPS1* (*BGIOSGA006694*), *OsGID2* (*BGIOSGA008496*), and *OsPP2C* (*BGIOSGA017524*); three genes involved in carbon metabolism, namely, *OsADH* (*BGIOSGA017928*), *OsIDH* (*BGIOSGA020406*), and *OsMDH* (*BGIOSGA017515*); four genes related to starch and sucrose metabolism, namely, Os*α-AMY* (*BGIOSGA003500*), *OsCx* (*BGIOSGA021512*), *Osβ-GC* (*BGIOSGA014893*), and *OsTPP* (*BGIOSGA005896*); and two genes involved in phenylpropanoid biosynthesis, namely, *OsPAL* (*BGIOSGA005999*) and *Os4CL* (*BGIOSGA026897*). As shown in Fig. 9, the expression pattern of all 12 DEGs measured by qRT‒PCR was consistent with the results of RNA-Seq analysis, demonstrating that the RNA-Seq data in the current study were credible and could further support the transcriptomic analysis presented above.

## Discussion

PHS causes severe yield and end-use quality losses in cereal crops worldwide, especially under high humidity conditions [28]. It is a complex biological process involving numerous physiological changes regulated by many genes [1]. In recent years, greater attention has been given to elucidating the mechanisms of PHS tolerance for breeding PHS-resistant varieties of rice and other cereal crops. In this study, we simulated high humidity conditions by spraying water on rice panicles at the ripening stage. We observed a great difference in PHS resistance between the two conventional rice varieties tested (JXZ and MXXZ). Under high humidity conditions, JXZ had a higher occurrence of PHS than MXXZ, demonstrating that MXXZ was more tolerant to PHS than JXZ (Fig. 1). We further characterized the physiological responses and transcriptomic expression profiles of the seeds of the two rice varieties under high-humidity treatment (Fig. 2, 3). The results showed that the number of DEGs was higher in PHS-sensitive JXZ than in PHS-resistant MXXZ. Additionally, a higher number of upregulated genes was identified in JXZ than in MXXZ, while more downregulated genes were identified in MXXZ than in JXZ (Fig. 3A). These results suggested that the physiological and biochemical processes in PHS-sensitive JXZ were more activated than those in MXXZ. Moreover, KEGG pathway analysis showed that plant hormone signal transduction, carbon metabolism, starch and sucrose metabolism, and phenylpropanoid biosynthesis were significantly enriched pathways (Fig. 3C, D). This indicates that these pathways play an important role in promoting PHS under high-humidity treatment.

Seed dormancy occurs during the late maturation stage of seed development. It is an important agricultural trait in many cereals, as it is linked to PHS [4, 29]. Crop seeds require a well-defined balance between germination potency and dormancy to avoid germination under unfavourable conditions. ABA is an important phytohormone that promotes seed dormancy, whereas GA breaks dormancy and promotes seed germination [8, 10]. Studies using GA and ABA biosynthesis and signalling mutants revealed their antagonistic roles in dormancy and germination. Changes in their endogenous levels and their respective signalling pathways are closely correlated with either maintaining dormancy or promoting germination [5, 30]. According to KEGG analysis, plant hormone signal transduction pathways were the most overrepresented in our study (Fig. 3C, D). We found that the expression of most genes (*CPS*, *KS*, *KAO*, *GA20ox*, and *GA2ox*) encoding key enzymes of GA biosynthesis and genes (*GID1*, *GID2*, and *DELLA*) involved in the GA signalling pathway were upregulated in both JXZ and MXXZ varieties. Compared with MXXZ, the PHS-sensitive JXZ had a larger number of upregulated genes encoding GA biosynthetic enzymes and signal transduction-related genes (Fig. 5; Table S9), indicating greater activation in JXZ, consistent with phenotypical and physiological studies (Fig. 1, 2). An increase in ABA degradation along with the attenuation of ABA signalling in seeds has been reported to promote seed germination under favourable conditions [31]. Os*ABA8ox*, encoding ABA 8'-hydroxylase in rice, is a hydroxylase involved in the oxidative degradation of ABA and is considered the pivotal enzyme for ABA inactivation [32]. Our results showed that the expression of *ABA8ox* was upregulated in JXZ, while it remained unchanged in MXXZ (Fig. 5; Table S9), suggesting that the degradation of ABA occurred more quickly in the seeds of JXZ than in MXXZ as PHS proceeded. The ABI5 transcription factor plays a crucial role in ABA signalling during seed germination. In the model plant *Arabidopsis thaliana*, disruption of *ABI5* decreases the sensitivity of seed germination to ABA inhibition [33]. We identified an *OsABI5-like* gene that exhibited significant downregulation in both varieties, suggesting that ABI5-mediated ABA signalling is attenuated when PHS occurs (Fig. 5; Table S9). HDA6 and BBX21 are two important negative regulators of ABA inhibition of seed germination in Arabidopsis [34, 35]; thus, their action promotes germination. We showed that the genes encoding HDA6 and BBX21 were consistently upregulated in PHS-sensitive JXZ but remained unchanged in MXXZ (Fig. 5; Table S9). This suggests that their expression levels could be an important reference parameter for evaluating the degree of PHS resistance among different rice varieties. Further studies on HDA6 and BBX21 biological functions related to PHS are needed in the future.

The phytohormone GA regulates seed germination mainly by affecting carbohydrate metabolism. During seed germination in cereal crops, GA induces α-amylase gene expression and its biosynthesis. α-amylase is then secreted into the endosperm to catalyse the hydrolysis of starch into soluble sugars [36–38]. Thus, it can be speculated that PHS in rice may exert a similar effect on starch mobilization through similar mechanisms based on the following experimental evidence. First, more upregulated genes encoding GA biosynthetic enzymes and signal transduction-related components were identified in PHS-sensitive JXZ than in PHS-resistant MXXZ (Fig. 5; Table S9). Second, PHS induced the expression of most amylase-encoding genes in both varieties. Moreover, the number of upregulated amylase genes was higher in JXZ than in MXXZ, while the number of downregulated amylase genes was higher in MXXZ than in JXZ (Fig. 7; Table S11). Third, in both varieties, PHS caused a significant decrease in starch content but a significant increase in glucose and total amylase activity. These physiological parameters changed to a much greater extent in JXZ than in MXXZ (Fig. 2). INV and α-GC are important enzymes involved in sucrose metabolism. INV catalyses the conversion of sucrose into fructose, and maltose is hydrolysed to glucose by α-GC [39,40]. Wakabayashi et al. (2015). reported that sucrose might be a metabolic intermediate, and the decrease in sucrose decomposition leads to low levels of glucose and fructose and thus inhibits seed germination [41]. The current study found that all the genes encoding INV and α-GC were significantly upregulated in the PHS-sensitive variety JXZ. However, their expression remained unchanged except for a shared DEG encoding α-GC, which was upregulated in MXXZ (Fig. 7; Table S11). Accordingly, the glucose content in JXZ is higher than that in MXXZ when PHS occurs (Fig. 2B). These results suggest that the lower glucose contents in MXXZ might give the tolerant genotype more capability to resist PHS than JXZ. Cellulose is a polysaccharide and the major structural component of plant cell walls. Cx hydrolyses cellulose to produce cellodextrin or cellobiose, and then β-GC hydrolyses these substances to glucose [42]. At the initial stage of crop seed germination, the cellulose in the endosperm cell wall is decomposed from Cx and β-GC, which loosens the entire endosperm structure and leads to endosperm liquefaction [42]. We found that most genes encoding Cx and β-GC exhibited a similar expression trend (upregulated) in both varieties. Nevertheless, they were more highly expressed in JXZ than in MXXZ (Fig. 7; Table S11). These findings indicate that the sensitivity to PHS of rice varieties might be closely associated with the changes in the cell wall composition and the activity of the enzymes involved in cell wall decomposition.

It has been commonly assumed that PHS is an energy-demanding process. Glycolysis and the TCA cycle are two fundamental processes in higher plants that produce energy. GAPDH, an important enzyme in glycolysis, catalyses the conversion of glyceraldehyde-3-phosphate to 1,3-bisphosphoglycerate [43]. In the model plant Arabidopsis, GAPDH was found to accumulate in nondormant seeds but not in dormant seeds, and a large number of enzymes involved in cellular energetic metabolism accumulated upon dormancy release of seeds [44, 45]. Nonogaki et al. (2010) and Weitbrecht et al. (2011) also assumed that activation of respiration is an early key event for seed germination [46, 47]. This accumulated evidence indicates that the breaking of seed dormancy is an energy-dependent process in higher plants. In most grain seeds, energy supply is mainly through transcriptional upregulation of genes involved in glycolysis, which catalyse the production of pyruvate [48]. Pyruvate then enters the TCA cycle and provides energy for seed germination [49]. In the current study, we found that most gene expression changes involved in glycolysis showed the same trend (upregulated) in both varieties. The number of upregulated genes was greater in JXZ than in MXXZ (Fig. 6; Table S10). In addition, the genes encoding ADH, involved in alcohol metabolism under anaerobic conditions, were significantly more upregulated in JXZ, while the expression levels of most *ADH* genes remained unchanged in MXXZ (Fig. 6; Table S10). Under aerobic conditions, pyruvate produced by glycolysis enters the TCA cycle, and more ATP is produced. The number of upregulated genes involved in the TCA cycle was higher in JXZ than in MXXZ. In contrast, the number of downregulated genes was higher in MXXZ than in JXZ (Fig. 6; Table S10). These results suggest that the energy produced by glycolysis and the TCA cycle possibly plays an essential role in the promotion of PHS in rice. The sensitivity of JXZ and tolerance of MXXZ to PHS might be attributed to the higher expression level of the genes encoding enzymes involved in the above two basic metabolic processes related to energy production in JXZ compared to MXXZ.

Phenylpropanoid biosynthesis is a complex secondary metabolism pathway, and the enzymes involved include PAL, C4H, 4CL, HCT, C3'H, CCoAOMT, CCR, CAD, F5H, COMT, and PRX [50, 51]. PAL is the first functional enzyme of the phenylpropanoid pathway that catalyses the conversion of phenylalanine to cinnamic acid, a precursor of lignin and flavonoids. C4H and 4CL catalyse the cinnamic acid to *p*-commaroyl-CoA conversion, which is then converted into lignin by CCR, CAD, and PRX. Changes in the gene expression encoding these enzymes can affect lignin and flavonoid accumulation [52–54]. According to our KEGG analysis data, "phenylpropanoid biosynthesis" was one of the most enriched KEGG pathways that were identified explicitly in JXZ (Fig. 3C). Considering that JXZ is a PHS-sensitive variety, it is speculated that the altered expression of genes involved in phenylpropanoid biosynthesis is important for developing PHS in rice. Further DEG analysis showed that the expression of enzymes involved in lignin biosynthesis in both varieties was upregulated, and the number of upregulated genes in JXZ was higher than that in MXXZ (Fig. 8; Table S12). Since lignin and flavonoids are two of the most important secondary metabolites, we speculated that the metabolite flow of the phenylpropanoid biosynthesis pathway was mainly to enhance lignin biosynthesis and reduce flavonoid biosynthesis during the process of PHS, which might have been beneficial to the PHS of rice. This is consistent with the impaired flavonoid metabolism in Arabidopsis mutants, which exhibit a reduction in seed dormancy, leading to better germination performance under optimal conditions [55, 56]. In the cereal crop wheat, it was also found that strong dormancy is closely related to a red seed coat colour (red seed coats contain a higher content of anthocyanin, which belongs to the flavonoid). In contrast, the genotypes with white seed coats (white seed coats contain lower anthocyanin content) are weakly dormant and thus susceptible to PHS damage [57, 58]. Therefore, an important research direction in the future to control the PHS of rice could be by manipulating the expression of key genes involved in the phenylpropanoid biosynthesis pathway.

## Conclusions

In summary, we provided novel insight into the physiological and molecular changes between PHS-sensitive and PHS-resistant rice varieties under high humidity conditions. Enrichment and expression pattern analysis indicated that the promotion of PHS in rice resulted from altered phytohormone regulation, more active carbon metabolism and energy production, and enhanced phenylpropanoid biosynthesis. The results from physiological data analysis further supported these observations. Based on the comparative transcriptome analysis, we speculate that some genes identified in our study may play a crucial role in the regulation of PHS in rice, including genes involved in phytohormone metabolism and signalling (such as *KAO* and *NCED3*), carbon metabolism (such as *α-AMY1.1* and *β-GC29*), and phenylpropanoid biosynthesis (such as *CAD3* and *PRX2*). These candidate genes will provide an important reference for future studies of the molecular mechanisms of PHS and breeding for PHS-resistant rice varieties through molecular breeding approaches.

## Methods

### Plant material and growth conditions

Two *indica* rice varieties were used in the experiments: the PHS-sensitive variety Jiuxiangzhan (JXZ) and the PHS-resistant variety Meixiangxinzhan (MXXZ). The tub-planting method was used to culture rice, as described previously [59]. Briefly, pre-germinated seeds were sown onto wet (saturated) paddy soils at the experimental site located at the Science and Technology Park of Jiangxi Agricultural University. After three weeks, the seedlings were transplanted into pots with four seedlings per pot. Each pot (length, 27.5 cm; width, 21.0 cm; height, 32.0 cm) contained 8.0 kg of soil. Before seedlings were transplanted into pots, 1.3 g urea, 5.0 g calcium-magnesium phosphate fertilizer and 1.4 g potassium chloride were applied to each pot. At the tillering stage, 0.5 g urea was supplemented in each pot. At panicle initiation, 0.8 g urea and 0.6 g potassium chloride were also applied to each pot. Pests, diseases, and weeds were intensively controlled in accordance with local recommendations for high yield production. To ensure that only uniformly developed samples would be used for analysis, rice panicles with the same heading date were tagged. On the 35^th^ day after heading, the tub-grown rice plants with the same tag were transferred to chambers and maintained at a temperature of 30.0 ± 1.0 °C during the light period (12 h) and 25.0 ± 1.0 °C during the dark period (12 h). Subsequently, the panicles of approximately 40 rice plants in the experimental group (high-humidity treatment) were sprayed with 2.0 L of distilled water at 2-h intervals for 5 days to maintain high humidity (100% relative humidity), while the panicles of rice plants in the control group were left untreated (75% relative humidity). For the PHS assay, the number of germinated seeds was counted every 24 h for 5 days. Germination was indicated by radicle growth from the seed. For transcriptomic analysis and determination of physiological parameters, the seeds in different panicles of the two rice varieties were sampled 48 h after spraying with distilled water or not immediately frozen in liquid nitrogen and stored at − 80 °C until further use.

### Determination of starch, glucose and soluble sugar contents

For starch determination, approximately 0.1 g of each sample was homogenized in 1.0 mL of extraction reagent (80% ethanol). The homogenized sample was then transferred into a centrifuge tube and incubated at 80 °C in a water bath for 30 min. Next, the sample was centrifuged for 5 min. The precipitate was added to 0.5 mL distilled water and incubated at 95 °C in a water bath for 15 min. After cooling, 0.35 mL of the reagent (HClO_4_) was added, and the sample was left to stand for 15 min at room temperature. The sample was then centrifuged at 3000 × g for 10 min at 25 °C. After centrifugation, a total of 0.4 mL supernatant was transferred to a new tube, and an equal volume of distilled water was added, mixed fairly vigorously, and used for the determination of starch content. For glucose determination, approximately 0.1 g of each sample was homogenized in 1.0 mL of distilled water. The homogenized sample was then transferred into a centrifuge tube and incubated at 95 °C in a water bath for 10 min. After cooling, the extraction was then centrifuged at 8000 × g for 10 min at 25 °C, and the supernatant was used for the determination of glucose content. For soluble sugar determination, approximately 0.1 g of each sample was added to 1.0 mL of distilled water and homogenized for 30 s. The homogenized solution was then transferred into a centrifuge tube and incubated at 95 °C in a water bath for 10 min. Next, the sample was centrifuged at 8000 × g for 10 min at 25 °C, and the supernatant was diluted tenfold with distilled water before analysis. The starch, glucose and soluble sugars were detected and calculated using the corresponding detection kits (Suzhou Michy Biomedical Technology Co., Ltd., China) according to the manufacturer’s protocol.

### Determination of amylase activity

To determine amylase activity, approximately 0.1 g of each sample was homogenized in 1.0 mL of distilled water, and the homogenized sample was then transferred into a centrifuge tube. Next, the tubes were shaken every 5 min for 15 min at room temperature, and the samples were centrifuged for 10 min. After centrifugation, the supernatant was diluted tenfold with distilled water before analysis. The α-amylase and total amylase (α-amylase + β-amylase) activities were detected and calculated using an amylase activity detection kit (Suzhou Michy Biomedical Technology Co., Ltd., China) according to the manufacturer's protocol. The activity of β-amylase was calculated as the difference between the total amylase activity and the α-amylase activity.

### RNA extraction, library construction and transcriptome sequencing

Total RNA was extracted from the 12 samples (JXZ-C1, JXZ-C2, JXZ-C3, JXZ-T1, JXZ-T2, JXZ-T3, MXXZ-C1, MXXZ-C2, MXXZ-C3, MXXZ-T1, MXXZ-T2 and MXXZ-T3) using an EASYspin Plant RNA Extraction kit (RN40, Aidlab, China), and the contaminating genomic DNA was removed using the RNeasy® MinElute® Cleanup Kit (Qiagen, Germany) following the manufacturer's instructions. The RNA concentration and purity were determined using an Agilent 2100 Bioanalyzer (Agilent Technologies, Inc., Santa Clara, CA, USA). All cDNA libraries were constructed using NEBNext Ultra RNA Library Prep Kit for Illumina (NEB, E7530) and NEBNext Multiplex Oligos for Illumina (NEB, E7500) according to the directions of the manufacturer. The cDNA libraries were then sequenced through the Illumina NovaSeq 6000 platform (Illumina, San Diego, CA). Briefly, the enriched mRNA was fragmented into approximately 200 nt RNA inserts, which were used to synthesize first-strand cDNA and second-strand cDNA. The double-stranded cDNA was subjected to end-repair and adaptor ligation. The suitable fragments were isolated by Agencourt AMPure XP beads (Beckman Coulter, Inc. and enriched by PCR amplification.

### Transcriptome analysis using reference genome-based read mapping

Before assembly, adaptor sequences, empty reads, and low-quality sequences were removed by Perl script. The clean reads filtered form raw reads were mapped to the *Oryza sativa* reference genome (*Oryza indica.* ASM465v1_release 49) using Tophat2 software [60]. The aligned records were then examined to remove potential duplicate molecules.

After removing the low-quality reads and adapter sequences, a total of 252 million clean reads were generated, representing an average of 20.96 million clean reads per sample. A total of 92.5% of the reads were mapped to the rice genome, with at least 88.4% of the clean reads being mapped to unique regions. The average GC content and Q30 of the 12 samples were 51.9% and 93.4%, respectively (Table S13). There was a strong correlation (correlation coefficient ≥ 0.80) between the biological replicates, suggesting that our RNA-Seq data were reliable for downstream analyses (Fig. S1).

### Identification of differentially expressed genes

Differential expression analysis of each comparison was conducted using the DESeq2 R package [61]. The gene expression differences between the samples were calculated based on the ratio of the FPKM (Fragments Per Kilobase of transcript per Million fragments mapped) values by the Cufflinks software [62]. In the current study, genes with a fold change value ≥ 2 (|Log_2_ fold change|≥ 1) between the treatment and control groups and with an adjusted FDR (false discovery rate) significance score < 0.01 were considered to be differentially expressed.

### Gene Ontology and Kyoto Encyclopedia of Genes and Genomes (KEGG) enrichment analysis

GO enrichment analysis of the DEGs was implemented using the software method for GOseq based on the Wallenius noncentral hypergeometric distribution [63]. KEGG is a database resource for understanding gene functions and analysing genome information [64–66]. KEGG, as a major public database of pathways, provides all possible metabolic pathways and associated annotations. In our study, the KOBAS 2.0 software was adopted to test the statistical enrichment of differential expression genes in KEGG pathways [67].

### Validation of RNA-Seq by qRT‒PCR assay

Total RNA was extracted from rice seeds using RNAiso Plus reagent (TaKaRa, China). First-strand cDNA was synthesized as described in a previous report [68]. For real-time quantitative PCR analysis, the reaction was performed using Hieff qPCR SYBR Green Master mix (YEASEN, Shanghai, China) on a CFX96 (Bio-Rad) following the manufacturer’s instructions. The conditions were as follows: denaturation for 2 min at 95 °C, followed by 40 cycles of 95 °C for 10 s and 60 °C for 30 s. Gene expression was normalized to that of *OsUBQ* by subtracting the C_T_ value of *OsUBQ* from the C_T_ value of the gene of interest. Expression ratios were then obtained from the Eq. 2^ΔΔCT^. The primer sequences used for qRT‒PCR analysis are shown in Table S14.

### Statistical analysis

All experiments in the current study were repeated three times independently (except for RNA-Seq, which were performed once). RNA obtained from the three biological replicates were pooled and separated in three analytical replicates for RNA-Seq analysis. The data presented in this study are mean values with standard errors (mean ± SE). The significant differences between the control and treatment of the samples were analysed by Student’s *t* test. The results with *P* < 0.05 were considered statistically significant and denoted by one star.

## Supplementary Information

**Additional file 1:** **Fig. S1.** Heatmap showing the results of pairwise correlation analyses between different samples.

**Additional file 2:** **Table S1.** The list of annotated DEGs identified in JXZ-T vs. JXZ-C.

**Additional file 3:** **Table S2.** The list of annotated DEGs identified in MXXZ-T vs. MXXZ-C.

**Additional file 4:** **Table S3.** The list of annotated DEGs identified in MXXZ-C vs. JXZ-C.

**Additional file 5:** **Table S4.** The list of annotated DEGs identified in MXXZ-T vs. JXZ-T.

**Additional file 6:** **Table S5.** The results of KEGG enrichment of DEGs in JXZ-T vs. JXZ-C.

**Additional file 7:** **Table S6.** The results of KEGG enrichment of DEGs in MXXZ-T vs. MXXZ-C.

**Additional file 8:** **Table S7.** Summary of GO results of the DEGs in JXZ-T vs. JXZ-C.

**Additional file 9:** **Table S8.** Summary of GO results of the DEGs in MXXZ-T vs. MXXZ-C.

**Additional file 10:** **Table S9.** The list of representative DEGs related to phytohormone metabolism and signalling.

**Additional file 11:** **Table S10.** The list of representative DEGs related to carbon metabolism (associated with energy production).

**Additional file 12:** **Table S11.** The list of representative DEGs related to starch and sucrose metabolism.

**Additional file 13:** **Table S12.** The list of representative DEGs related to phenylpropanoid biosynthesis.

**Additional file 14:** **Table S13.** The general information of sequencing reads and reads that mapped to the reference genome.

**Additional file 15:** **Table S14.** Primers used for quantitative real-time PCR in this study.

## Data Availability

All data generated or analyzed during this study are included in this published article and its supplementary information files. The raw RNA-Seq data in this manuscript are available for downloading from the NCBI Sequence Read Archive (BioProject ID: PRJNA866656).

## Abbreviations

4CL: 4-Coumarate CoA ligase
α-AMY: α-Amylase
AAO: ABA-aldehyde oxidase
ABA: Abscisic acid
ABA8ox: ABA 8ʹ-hydroxylases
ABI3: Abscisic acid insensitive 3
ABI5: Abscisic acid insensitive 5
ACO: Aconitase
α-GC: α-Glucosidase
β-AMY: β-Amylase
BBX21: B-box domain protein 21
β-GC: β-Glucosidase
C3'H: *p*-Coumarate 3-hydroxylase
C4H: Cinnamate 4-hydroxylase
CAD: Cinnamyl alcohol dehydrogenase
CCoAOMT: Caffeoyl-CoA O-methyltransferase
CCR: Cinnamoyl-CoA reductase
COMT: Caffeic acid 3-O-methyltransferase
CPS: Ent-Copalyl diphosphate synthase
CRTISO: Carotenoid isomerase
CS: Citrate synthase
Cx: Cellulase
DEGs: Differentially expressed genes
DLST: Dihydrolipoamide succinyltransferase
ENO: Enolase
F5H: Ferulate 5-hydroxylase
FBA: Fructose-bisphosphate aldolase
GA: Gibberellin acid
GA2ox: Gibberellin 2-β-dioxygenase
GA3ox: Gibberellin 3-oxidase
GA20ox: Gibberellin 20 oxidase
GAPDH: Glyceraldehyde-3-phosphate dehydrogenase
GID: Gibberellin insensitive dwarf
GO: Gene ontology
HCT: Hydroxycinnamoyl transferase
HDA6: Histone deacetylase 6
IDH: Isocitrate dehydrogenase
INV: Invertase
ISA: Isoamylase
KAO: Ent-Kaurenoic acid oxidase
KEGG: Kyoto encyclopedia of genes and genomes
KO: Ent-Kaurene oxidase
KS: Ent-Kaurene synthase
LCY: Lycopene β-cyclase
LSC: Succinyl-CoA synthetase
MDH: Malate dehydrogenase
NCED: 9-Cis-epoxycarotenoid dioxygenase
PAL: Phenylalanine ammonia lyase
PDHC: Pyruvate dehydrogenase complex
PDS: Phytoene desaturase
PGK: Phosphoglycerate kinase
PGM: Phosphoglycerate mutase
PHS: Pre-harvest sprouting
PK: Pyruvate kinase
PP2C: Protein phosphatase 2C
PRX: Peroxidase
PYR/PYL/RCAR: Pyrabactin resistance/pyrabactin-like/regulatory components of ABA receptors
SDH: Succinate dehydrogenase
SnRK2: SNF1-related protein kinase 2
TFs: Transcription factors
TPP: Trehalose 6-phosphate phosphatase
TPS: Trehalose 6-phosphate synthase
VDE: Violaxanthin de-epoxidase
ZDS: ζ-Carotene desaturase
ZEP: Zeaxanthin epoxidase
