# Supplementary material for: Comparative transcriptomic analysis provides insights into the molecular basis underlying pre-harvest sprouting in rice
Source: BMC Genomics. 2022 Nov 24;23:771. doi: 10.1186/s12864-022-08998-4 (PMC9701047; doi:10.1186/s12864-022-08998-4)
Supplement: Supplementary file 1 — Additional file 1: Fig. S1. Heatmap showing the results of pairwise correlation analyses between different samples. [file 12864_2022_8998_MOESM1_ESM.doc]

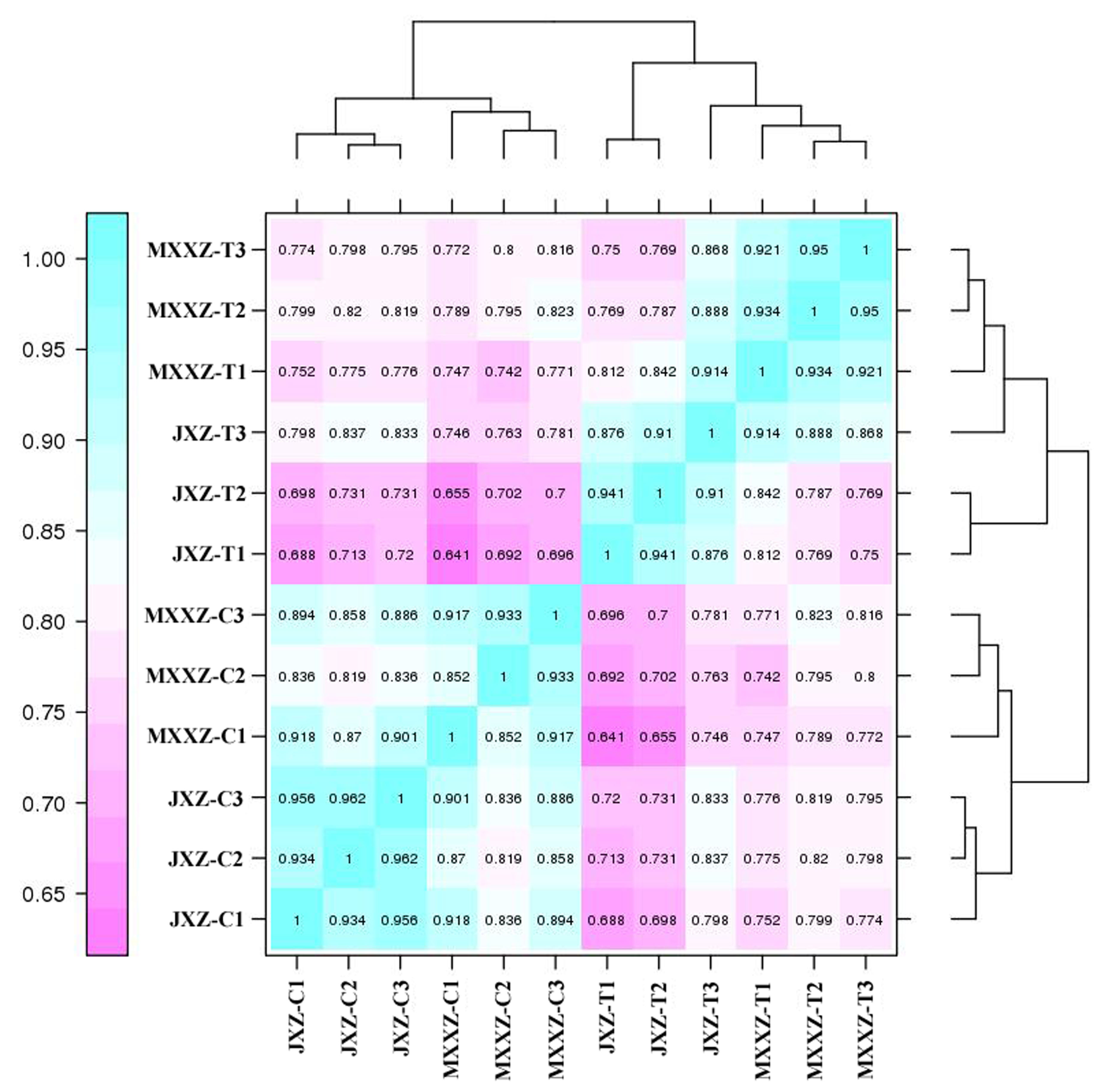


**Fig. S1.** Heatmap showing the results of pairwise correlation analyses between different samples. A value close to 1 indicates high similarity between different samples. “JXZ-C”, “JXZ-T”, “MXXZ-C” and “MXXZ-T” indicate the control (C) and PHS-induced (T) samples of the JXZ and MXXZ varieties, respectively; “1”, “2” and “3” indicate three biological replicates.
